# Hyperbaric Oxygen Therapy Can Induce Neuroplasticity and Significant Clinical Improvement in Patients Suffering From Fibromyalgia With a History of Childhood Sexual Abuse—Randomized Controlled Trial

**DOI:** 10.3389/fpsyg.2018.02495

**Published:** 2018-12-17

**Authors:** Amir Hadanny, Yair Bechor, Merav Catalogna, Shir Daphna–Tekoah, Tal Sigal, Mehrzad Cohenpour, Rachel Lev-Wiesel, Shai Efrati

**Affiliations:** ^1^Sagol Center for Hyperbaric Medicine and Research, Assaf Harofeh Medical Center, Zerifin, Israel; ^2^Galilee Faculty of Medicine, Bar Ilan University, Ramat Gan, Israel; ^3^Sackler School of Medicine, Tel-Aviv University, Tel-Aviv, Israel; ^4^The Emili Sagol CAT Research Center, University of Haifa, Haifa, Israel; ^5^Faculty of Social-Work, Ashkelon Academic College, Ashkelon, Israel; ^6^Social-Work Service, Kaplan Medical Center, Rehovot, Israel; ^7^Radiology Department, Assaf Harofeh Medical Center, Zerifin, Israel; ^8^Nuclear Medicine Institute, Assaf Harofeh Medical Center, Zerifin, Israel; ^9^Research and Development Unit, Assaf Harofeh Medical Center, Zerifin, Israel; ^10^Sagol School of Neuroscience, Tel-Aviv University, Tel-Aviv, Israel

**Keywords:** fibromyalgia, childhood sexual abuse, FMS, CSA, PTSD, post trauma, hyperbaric oxygen, HBOT

## Abstract

**Background:** Fibromyalgia syndrome (FMS), a condition considered to represent a prototype of central sensitization syndrome, can be induced by different triggers including childhood sexual abuse (CSA). Recent studies have demonstrated hyperbaric oxygen therapy (HBOT) can induce neuroplasticity and improve clinical outcome of FMS. The aim of the current study was to evaluate the effect of HBOT on patients suffering from FMS with a history of CSA.

**Materials and methods:** A prospective randomized clinical trial conducted between July 2015 and November 2017 included women with a history of CSA who fulfilled fibromyalgia diagnosis criteria for at least 5 years prior to inclusion. Included participants (*N* = 30) were randomly assigned to treatment group, treated with 60 HBOT sessions and a control/crossover group received psychotherapy. After the control period, the control/crossover group was crossed to HBOT. Clinical outcomes were assessed using FMS questioners, post-traumatic stress disorder (PTSD) questioners and quality of life questioners. Objective outcome were assessed using brain function and structure imaging.

**Findings:** Following HBOT, there was a significant improvement in all FMS questionnaires (widespread pain index, Fibromyalgia symptoms severity scale, Fibromyalgia functional impairment), most domains of quality of life, PTSD symptoms and psychological distress. The same significant improvements were demonstrated in the control following crossover to HBOT. Following HBOT, brain SPECT imaging demonstrated significant increase in brain activity in the prefrontal cortex, orbital frontal cortex, and subgenual area (*p* < 0.05). Brain microstructure improvement was seen by MRI-DTI in the anterior thalamic radiation (*p* = 0.0001), left Insula (*p* = 0.001), and the right Thalamus (*p* = 0.001).

**Conclusion:** HBOT induced significant clinical improvement that correlates with improved brain functionality and brain microstructure in CSA related FMS patients.

**Trial Registration:** www.Clinicaltrials.gov, identifier: NCT03376269. url: https://clinicaltrials.gov/show/NCT03376269

## Introduction

Fibromyalgia syndrome (FMS) is a condition considered to represent a prototype of central sensitization syndrome, characterized by chronic widespread pain along with symptoms of fatigue, non-restorative sleep and cognitive difficulties (Buskila, 2009; Clauw et al., 2011; Schmidt-Wilcke and Clauw, 2011; Ablin et al., 2016). It affects 2–4% of the population, with 9:1 female-to-male incidence ratio (Buskila, 2009; Clauw et al., 2011; Schmidt-Wilcke and Clauw, 2011). FMS can be induced by traumatic brain injury and certain infections, such as a viral illness, Lyme disease or severe emotional stress such as childhood sexual abuse (CSA) (Sarzi-Puttini et al., 2011; Schmidt-Wilcke and Clauw, 2011).

The prevalence of post-traumatic stress disorder in FMS patients range between 15 and 56%. Moreover, even higher rates for what concern post-traumatic stress spectrum symptoms, worsening the both symptoms and quality of life FMS patients (Sancassiani et al., 2017). It is estimated that 10–64% of FMS patients have history of CSA (Walker et al., 1997; Goldberg et al., 1999; Imbierowicz and Egle, 2003; Häuser et al., 2013). In fact, there is also a correlation between past trauma history and symptoms severity (Walker et al., 1997; McBeth et al., 1999) where patients who suffered CSA, report higher psychological stress, greater functional disability and poorer psychological adjustment (Taylor et al., 1995).

Several possible mechanisms have been suggested to be responsible for the health implications of CSA survivors (Irish et al., 2010). First, behavioral modifications such as substance abuse, tobacco use and risky sexual behavior. Second, the coping strategies the patients adapt may themselves be associated with health outcomes as well as hostility and psychological (depression, anxiety, dissociation). However, recent evidence suggests that CSA, as other types of severe post-traumatic stress disorder (PTSD), may induce biological, physiological and neuroanatomic mechanisms leading to structural and functional changes in regions of the brain responsible for the long standing unremitting nature of the syndrome (Schnurr and Green, 2004). Previous studies have showed early trauma in CSA induces chronic stress that may culminate in structural and functional changes in affective, limbic and prefrontal brain regions such as the amygdala, insula, anterior cingulate (ACC), and prefrontal cortex (PFC) (Liberzon and Sripada, 2008). FMS patients with or without history of CSA have elevated activity in the somatosensory cortex and reduced activity in the frontal, cingulate, medial temporal and cerebellar cortices(Guedj et al., 2007, 2008). These findings indicate that the pain in fibromyalgia results primarily from abnormalities in pain processing pathways (Guedj et al., 2007, 2008; Ablin et al., 2016). In a recent study, FMS with PTSD report more potentially traumatic events, avoidance symptoms, numbing, arousal, maladaptive coping and personality characteristics compared to FMS patients without PTSD (Conversano et al., 2018).

There is no efficient agreed upon therapy for FMS. Pharmacotherapy, aerobic exercises and cognitive behavioral therapies, consist of symptom management (Goldenberg, 2008; Matthey et al., 2013). Integrated programs based on these treatments have moderate pain alleviation with limited effectiveness (Goldenberg, 2008). Hyperbaric oxygen therapy (HBOT), the application of hyperbaric pressure in conjunction with increased oxygen content, has been shown in several clinical studies to have the capacity to induce neuroplasticity that leads to repair of persistent impaired brain functions even years after an acute injury (Boussi-Gross et al., 2013, 2015; Efrati et al., 2013; Efrati and Ben-Jacob, 2014; Hadanny et al., 2015; Tal et al., 2015, 2017; Hadanny and Efrati, 2016a,b). The mechanisms involved in brain repair include increased cerebral blood flow, improved mitochondrial function, cellular metabolism and stem cells recruitment and mobilization (Hadanny and Efrati, 2015). Despite the fact that HBOT for neurological disorders is still considered controversial, recent evidence evaluated its effectiveness in treating FMS; To date, two prospective randomized controlled trials have demonstrated the efficacy of HBOT in fibromyalgia (Yildiz et al., 2004; Efrati et al., 2015). The improvement after HBOT was demonstrated in all clinical aspects of FMS corresponded with brain metabolism/function changes. In those studies, FMS patients were included based on FMS symptoms and not based on the potential trigger for the FMS. In addition, in a recent case series, the application of HBOT in FMS patients initiated recovery of repressed memories, which were all CSA related (Efrati et al., 2018).

The aim of the current study was to evaluate the effect of HBOT on clinical outcome and brain activity and microstructure of CSA survivors who developed chronic unremitting FMS.

## Materials and Methods

The study was performed as a prospective randomized clinical trial conducted at the Sagol center for hyperbaric medicine and Research of Assaf-Harofeh Medical Center, Israel between November 2015 and November 2017. The protocol was approved by the Assaf Harofeh institutional review board (202/14) and registered in the US National Institute of Health Clinical Trails registry (NCT03376269). The registry was posted prior to the actual study start and recruitment. Due to a technical error which was noticed upon completion, the final publication release was at the study completion. All participants signed written informed consent prior to their inclusion.

### Participants

The study included women over 18 years old with a history of CSA who had already underwent psychotherapy for at least a year prior to their inclusion and fulfill fibromyalgia diagnosis criteria (according to the American college of rheumatology (ACR) 2010 diagnostic criteria Wolfe et al., 2010) for at least 5 years prior to their inclusion and exhausted all available therapeutic psychological and pharmacological interventions.

Exclusion criteria included pregnancy, chest pathology incompatible with HBOT, inner ear disease, claustrophobia, other neurological conditions and inability to sign informed consent. In addition, if preliminary psychologist interview suggested women are unstable for chronic daily sessions, they were excluded. Smoking was not allowed during the study.

### Protocol

After signing an informed consent form, participants underwent baseline evaluation which included medical history, physical examination, psychological interview, questionnaires, and brain imaging. Included participants were randomly assigned to two groups (1:1 randomization): a treatment group and a control/crossover group. The crossover approach was adopted to the known sham/placebo problem in HBOT studies (Efrati and Ben-Jacob, 2014). The different aspects related to the placebo issue and the cross-over design are discussed in Appendix-III in the supplementary materials.

Participants in the treated group were evaluated twice–at baseline and after 3 months of HBOT. Participants in the crossover group were evaluated three times: baseline, after 3 months control period in which the patients received psychological therapy, and after crossover with subsequent 3 months of HBOT. Intention to treat analysis was performed on all included patients.

### Study Endpoints

#### Questionnaires

The participants filled the following *fibromyalgia related questionnaires*; Widespread pain index (WPI), Fibromyalgia symptoms severity scale (SSS), Fibromyalgia functional impairment (FIQ); *Quality of life related questionnaires*; Short Form-36 (SF-36). *PTSD related questionnaires*; The Brief Symptom Inventory−18(BSI-18), The PTSD symptom scale interview (PSS-I) and the *Childhood Trauma Questionnaire (CTQ)*. The participants filled the questionnaires in an online blinded service more than 1 week (1–4 weeks) after the end of the HBOT protocol. All questionnaires language were in Hebrew as it was the native language of all participants.

#### Fibromyalgia Related Symptoms Questionnaires

Widespread pain index (WPI)—a count of number of painful body regions. The WPI ranges 0–19 (Wolfe et al., 2010).

Fibromyalgia symptoms severity scale (SSS) (Wolfe et al., 2011)–a measure of three major symptoms (fatigue, trouble thinking or remembering, waking up tired [unrefreshed]) which can be coded 0–3 (0 = not present to 3 = extreme) and three additional symptoms (Pain or cramps in lower abdomen, depression, headache), which can be coded to be present (1) or not present (0) (total score 0–3). The SSS ranges from 0 to 12.

Fibromyalgia criteria diagnosis served as primary endpoint: either (a) WPI score ≥ 7 and SSS ≥ 5 or (b) WPI ≥ 3–6 and SSS ≥ 9 used as a cutoff for FMS diagnostic criteria according to the ACR (Wolfe et al., 2010).

Fibromyalgia functional impairment (Fibromyalgia Impact Questionnaire—FIQ)(Buskila and Neumann, 1996) designed to measure the components of health status most affected by FMS. The FIQ is composed of 10 items. The first item contains 11 questions related to physical functioning—each question is rated on a 4 point Likert type scale. Items 2 and 3 ask the patient to mark the number of days they felt well and the number of days they were unable to work (including housework) because of fibromyalgia symptoms. Items 4 through 10 are horizontal linear scales marked in 10 increments on which the patient rates work difficulty, pain, fatigue, morning tiredness, stiffness, anxiety, and depression. FIQ score ranges between 0 and 100.

#### Quality of Life Related Questionnaire

The RAND Health Status Survey, Short Form-36 (SF-36) was used to assess quality of life. RAND SF-36 is a self-report measure that evaluates physical functioning; bodily pain; role limitations due to physical health problems; role limitations due to personal or emotional health; general mental health; social functioning; energy/fatigue; and general health perception (McHorney et al., 1993, 1994; Russo et al., 1998). Each scale generates a score from 0 to 100, with a high score indicating better health and less body pain.

#### PTSD Related Questionnaires

The Brief Symptom Inventory−18 (BSI-18) was used to evaluate psychological distress (Recklitis et al., 2006). The BSI-18 is an 18 item self-report questionnaire which generates a summary scale, the global stress index (GSI), and three subscales: depression, anxiety, and somatization. Each item is rated on a 5 point scale, with distress ratings ranging from 0 (not at all) to 4 (extremely). Each subdomain ranges from 0 to 24 and the total score/GSI score ranges from 0 to 72.

The PTSD symptom scale interview (PSS-I) is a 17-item semi-structured interview that assesses the presence and severity of DSM-IV PTSD symptoms related to a single identified traumatic event in individuals with a known trauma history (Edna et al., 1993). Each item is rated according to a combination of frequency and severity (from 0 = “not at all” to 3 = “5 or more times per week/very much”).

PTSD severity is determined by totaling the 17 PSS-I symptom ratings. Total Score ranges from 0 to 51, and divided to three domains: re-experiencing symptoms (0–15) hyperarousal (0–21) and avoidance (0–15).

#### CSA Related Questionnaire

The Childhood Trauma Questionnaire was developed as a screening tool for histories of abuse and neglect (Fink et al., 1995). The self-report includes a 28-item test that measures 5 types of maltreatment—emotional, physical, and sexual abuse, and emotional and physical neglect. Items are rated on a 5-point Likert scale ranging from Never True to Very Often True. Each subscale score ranges from 5 (no history of abuse or neglect) to 25 (very extreme history of abuse and neglect).

#### Brain SPECT

Brain single photon emission computed tomography (SPECT) was conducted with 925–1,110 MBq (25–30 mCi) of technetium-99m-methyl-cysteinate-dimmer (Tc-99m-ECD) at 40–60 min post injection using a dual detector gamma camera (ECAM or Symbia T, Siemens Medical Systems) equipped with high resolution collimators. Data was acquired in 3-degree steps and reconstructed iteratively with Chang method (μ = 0.12/cm) attenuation correction.

Regional cerebral blood flow change analysis was conducted by fusing pre- and post-treatment studies that were normalized to median brain activity. SPECT images were reoriented into Talairach space using NeuroGam (Segami Corporation) for identification of Brodmann cortical areas and in order to compute the mean perfusion in each Brodmann area (BA).

#### Brain SPECT Analysis

Changes in perfusion in all Brodmann areas for each subject were determined by calculating the percentage difference between post-period and pre/baseline-period divided by the pre/baseline-period perfusion.

Independent two tailed *t*-test was performed to compare the relative change between (a) the groups after first 3 months of HBOT/control (b) both groups after 3 months of HBOT. Dependent two tailed *t*-test was performed to compare the relative change in the control/crossover group after the control period (3 months) and post HBOT (6 months). Statistical significance was considered as *P* < 0.05 following false discovery rate (FDR) for multiple hypothesis testing (Storey, 2002).

#### Brain Magnetic Resonance (MRI)

Imaging was performed using a 3 Tesla system (MAGNETOM Skyra, Siemens Medical Solutions) with a multichannel head coil as a receiver coil. The MRI protocol included the following sequences: T2 weighted, T1 weighted, FLAIR, susceptibility weighted imaging (SWI), and diffusion tensor imaging (DTI). The post-HBOT and post-control period MRI were done more than 1 week (1–4 weeks) after the end of the HBOT protocol.

DTI protocol included: 30 diffusion weighted images were scanned with different gradient directions (*b* = 1,000) and one volume without diffusion weighting, with the following parameters: TR = 9,300 ms, TE = 91 ms, Voxel size = 1.5 × 1.5, Matrix = 150 × 150, No. of slices = 63, Slice thickness = 2.2 mm.

#### MRI Analysis

MRI analysis was performed by WiseImage (Hod Hasharon, Israel, www.wise-image.com). Motion and Echo planar imaging (EPI) correction and regularization of the DWI volumes as well as calculation of DTI maps (MD = mean diffusivity, FA = fractional anisotropy, AD = axial diffusivity, RD = radial diffusivity maps) were done using ExploreDTI software (Leemans et al., 2009). Using voxel-based analysis, generating statistical parametric maps. Voxel fractional anisotropy (FA) were measured in the white matter. FA delta values were calculated by subtracting two maps (before and after control period, before and after HBOT).Independent paired *t*-test were performed between the treatment group post HBOT compared to the crossover/control control period and the HBOT period.

### Hyperbaric Oxygen Treatment

Participants were treated in a multiplace chamber (HAUX-Life-Support GmbH) for 60 daily sessions, 5 days a week. Each session consisted of 90 min of exposure to 100% oxygen at 2 ATA with 5 min air breaks every 20 min.

### Psychological Therapy

Both control and HBOT groups were supported with psychological therapy. The psychological therapy included journal writing, drawings, daily correspondence with the therapist and 1 h weakly session with the therapist.

### Statistical Analysis

In addition to the MRI and SPECT analysis described above, continuous data were expressed as means ± standard deviations. The normal distribution for all variables was tested using the Kolmogorov-Smirnov test. Independent *t*-test were performed to compare variables between the two groups. Dependent *t*-test were performed to compare changes within groups. *P*-value1 reflects an independent *t*-test comparing baseline scores between groups. *P*-value2 reflects an independent *t*-test comparing post HBOT/control scores between groups. *P*-value3 reflects a dependent *t*-test comparing post HBOT/control within the same group. *P*-value4 reflect an independent *t*-test comparing post HBOT scores between groups. *P*-value5 reflect a dependent *t*-test comparing post HBOT to pre-HBOT scores in the control group.

Categorical data is expressed in numbers and percentages and compared by chi-square test. Univariate analysis was performed using Chi-Square/Fisher's exact test (where appropriate) or to identify significant variables (*P* < 0.05). Pearson's correlations were performed between perfusion change in Brodmann areas and the change in questionnaires scores before and after HBOT. The alpha level was set to 0.05. Data were statistically analyzed using SPSS software (version 22.0).

Sample size was based on the assumption that HBOT will induce a 40% change in FMS diagnosis in the treatment group where by change improvement of 10% in the control group. The sample size was calculated to provide 80% power with 0.05 alpha.

## Results

**Figure 1:**
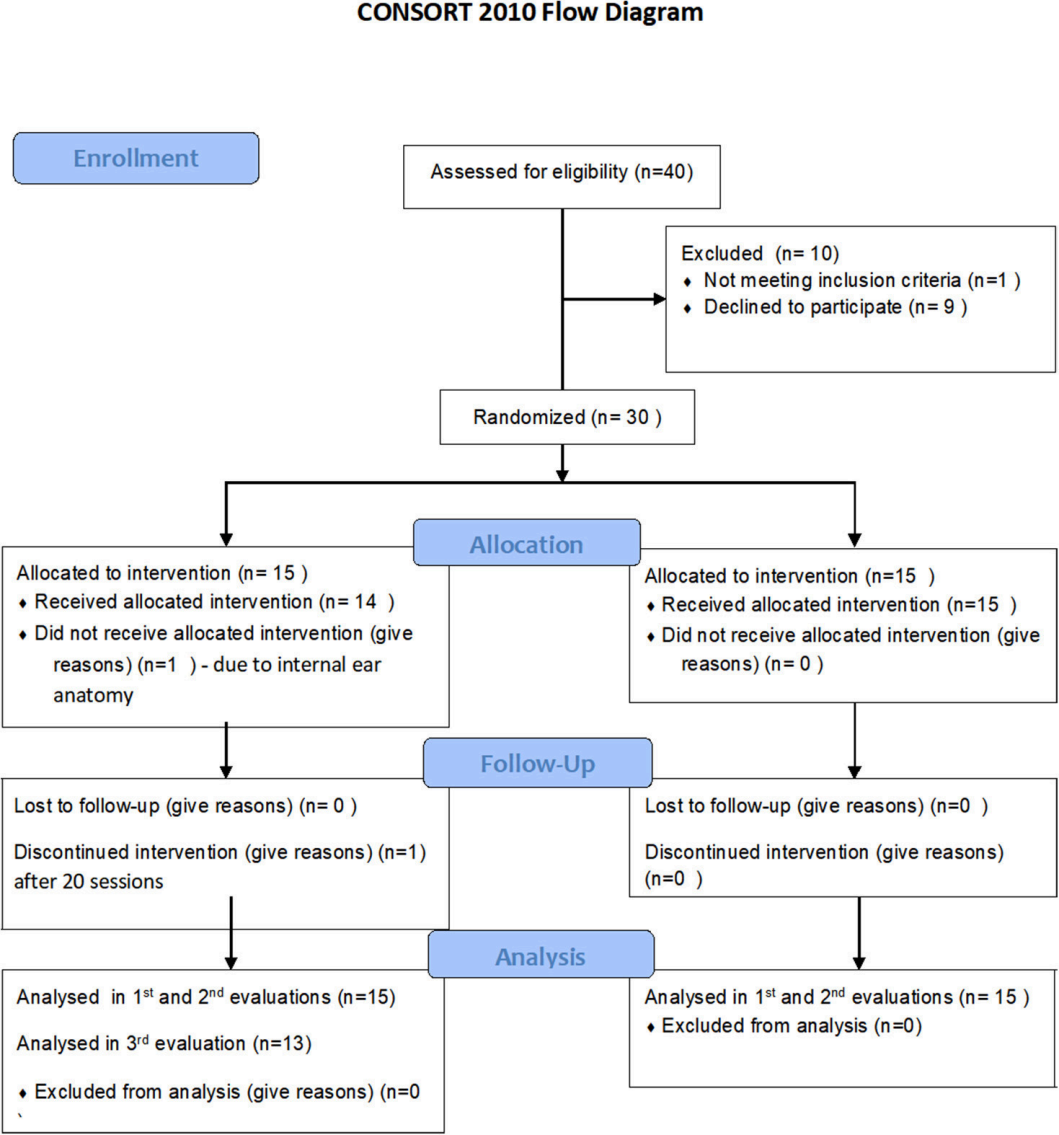
Participants flowchart.

Forty participants signed a written informed consent and randomized to either treatment or control/crossover groups. Ten participants were excluded prior to baseline evaluation (Figure 1). Thirty participants were included in the 2nd evaluation (post HBOT/control) and 28 participants in the 3rd evaluation (Figure 1). Participants' characteristics are summarized in Table 1. All participants were females age 45.9 ± 10.8, who suffered CSA at the age of 8.4 ± 4.5 years.

**Table 1:** Baseline characteristics.

		**Total (*N* = 30)**	**Treatment (*N* = 15)**	**Control (*N* = 15)**	**Sig**.
Age		45.9 ± 10.8	48.3 ± 10.6	43.1 ± 10.6	*P =* 0.15
Assault age		8.4 ± 4.5	9.1 ± 4.9	7.8 ± 4.1	*P =* 0.44
Assault duration					
	Single event	5 (16.7%)	3 (20%)	2 (13.3%)	*P =* 0.88
	Weeks	5 (16.7%)	3 (20%)	2 (13.3%)	
	Months	4 (13.3%)	2 (13.3%)	2 (13.3%)	
	Years	16 (53.3%)	7 (46.7%)	9 (60%)	
Identity of abuser					*P =* 0.51
	Parent	9 (30%)	5 (33.3%)	4 (26.7%)	
	Family	11 (36.7%)	4 (26.7%)	7 (46.7%)	
	Stranger	10 (33.3%)	6 (40.0%)	4 (26.7%)	
Education years		16.5 ± 3.3	16.9 ± 3.9	16.1 ± 2.8	*P =* 0.51
Marital status					*P =* 0.17
	Married	18 (60%)	8 (53.2%)	10 (55.6%)	
	Divorced	6 (20%)	5 (33.3%)	1 (6.7%)	
	Single	6 (20%)	2 (13.3%)	4 (26.7%)	
Children		2.2 ± 1.1	2.3 ± 1.1	2.1 ± 1.1	*P =* 0.66
Religion					*P =* 0.72
	Secular	19 (63.3%)	11 (73.3%)	8 (53.3%)	
	Traditional	5 (16.7%)	2 (13.3%)	3 (20%)	
	Religious	6 (20%)	2 (13.4%)	4 (26.7%)	
Employed		22 (73.3%)	12 (80.0%)	10 (66.7%)	*P =* 0.68 (F)
Disease related drugs					
	Baseline	15 (48.4%)	8 (53.3%)	7 (46.7%)	*P =* 0.59
	Post 3 months Treatment/Control	13 (41.9%)	5 (33.3%)	8 (50.0%)	*P =* 0.35
Anti-Depression Tx.		11 (36.7%)	6 (40%)	5 (33.3%)	*P =* 0.70
Previous suicide attempt		5 (16.7%)	3 (20%)	2 (13.3%)	*P* > 0.99(F)

### Effect on FMS Symptoms

**Figure 2:**
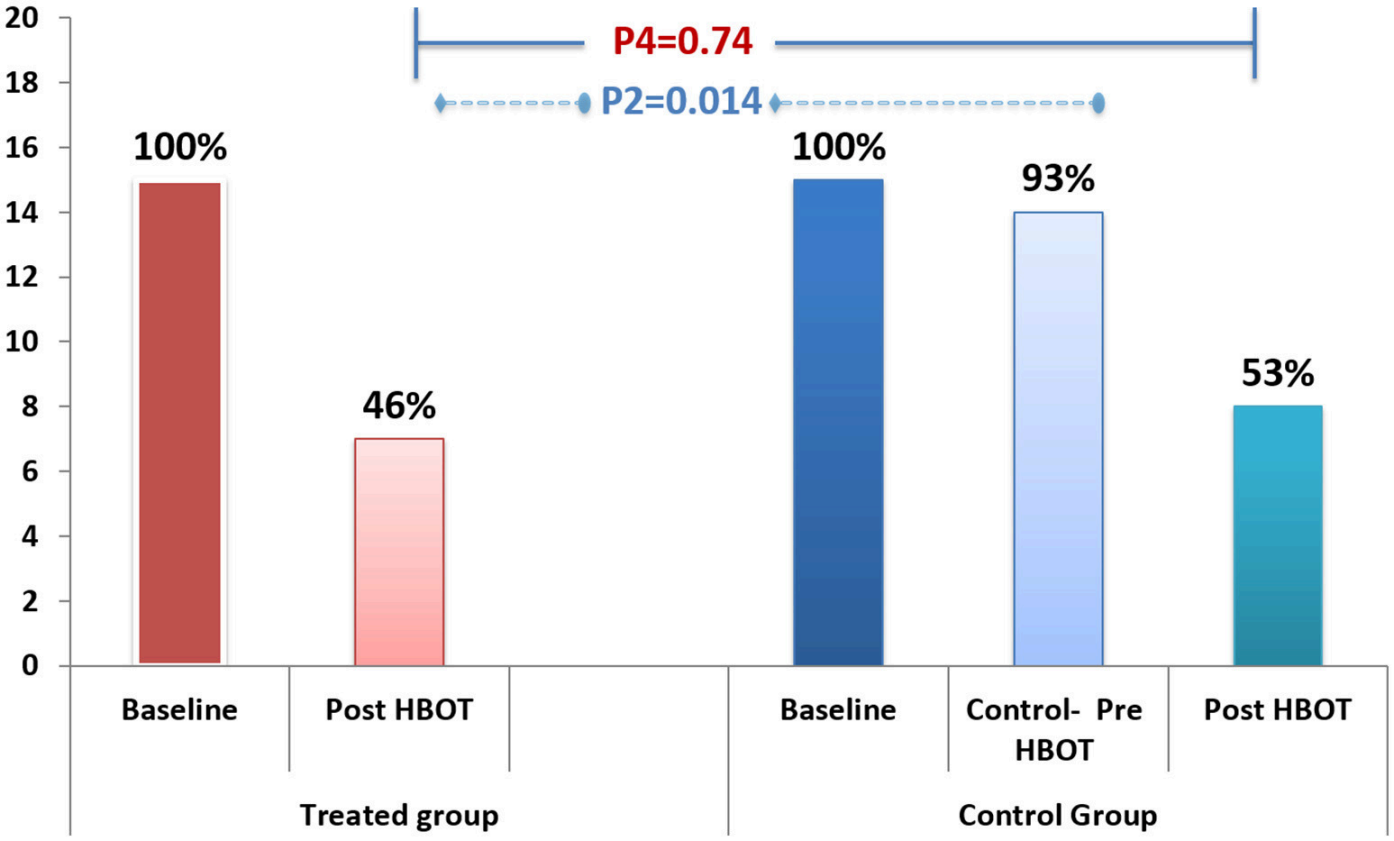
The effect of HBOT on FMS diagnosis.

Fibromyalgia criteria diagnosis: After HBOT, 53.3% recovered and did not fulfill FMS diagnosis criteria, compared to 6.7% in the control group during the control period (*P* = 0.014). After crossover and HBOT, 46% recovered. The recovery from FMS was similar in both groups following HBOT (Figure 2; Table 2).

**Table 2:** FMS questionnaires scores at baseline, post HBOT/control period and post crossover period.

	**Treated group (*****N*** **=** **15)**						**Control group (*****N*** **=** **15)**				**Crossed control group (*****N*** **=** **13)**			
	**Baseline**	**Post HBOT**	**P1**	**P2**	**Mean change**	**P3**	**Baseline**	**Control- Pre HBOT**	**Mean change**	**P3**	**Post HBOT**	**P4**	**Mean change**	**P5**
Tender point count (of 18)	11.6 ± 4.8	7.9 ± 5.4	0.967	**0.026**	(−3.7) ± 3.0	**<0.001**	11.6 ± 3.8	12.1 ± 4.2	0.5 ± 3.2	0.58	8.3 ± 4.5	0.85	(−3.7) ± 5.2	**0.02**
Symptom severity score	8.93 ± 2.1	4.93 ± 2.6	0.251	**<0.001**	(−4.0) ± 3.1	**<0.001**	9.7 ± 1.6	9.6 ± 1.9	(−0.07) ± 2.1	0.9	8.4 ± 2.4	**0.001**	(−1.1) ± 3.1	0.23
FMS diagnosis	15(100%)	7 (46.7%)	1 (F)	**0.014 (F)**			15 (100%)	14 (93.3%)			8 (53.3%)	0.71		
**QUALITY OF LIFE (SF-36)**														
*Physical functioning*	60.0 ± 30.0	70.3 ± 25.6	0.53	0.08	10.3 ± 15.7	**0.024**	54.0 ± 21.6	54.3 ± 22.7	0.3 ± 14.2	0.93	67.3 ± 28.0	0.77	14.6 ± 14.1	**0.003**
*Physical limitations*	21.7 ± 35.2	55.0 ± 42.5	0.48	**<0.001**	33.3 ± 34.9	**0.002**	13.3 ± 28.1	16.7 ± 27.8	3.3 ± 36.4	0.73	50.0 ± 43.3	0.76	34.6 ± 40.2	**0.009**
*Emotional limitations*	28.9 ± 30.5	55.5 ± 43.0	0.86	**0.027**	26.7 ± 42.2	**0.028**	26.6 ± 38.2	22.2 ± 34.9	(−4.4) ± 30.5	0.58	56.4 ± 39.4	0.95	33.3 ± 66.6	0.09
*Energy*	26.3 ± 16.1	48.3 ± 23.7	0.87	**0.003**	22.0 ± 21.3	**0.001**	25.3 ± 16.0	24.7 ± 14.3	(−0.6) ± 11.8	0.83	55.4 ± 27.9	0.48	30.8 ± 17.4	**<0.001**
*Emotional wellbeing*	45.1 ± 19.4	65.6 ± 17.5	0.93	**0.029**	20.5 ± 21.8	**0.003**	45.6 ± 13.2	51.2 ± 16.7	5.6 ± 15.4	0.18	67.4 ± 18.7	0.8	14.8 ± 20.6	**0.02**
*Social function*	36.7 ± 24.8	60.8 ± 31.3	0.63	**0.011**	24.2 ± 33.9	**0.015**	34.2 ± 20.8	36.7 ± 22.6	(−6.6) ± 18.2	0.18	71.1 ± 23.0	0.33	35.6 ± 19.7	**<0.001**
*Pain Domain*	34.7 ± 26.5	63.3 ± 30.0	0.58	**0.002**	28.7 ± 26.0	**0.001**	29.5 ± 24.7	32.5 ± 17.1	3.0 ± 20.9	0.59	31.2 ± 12.0	**0**	(−1.9) ± 18.7	0.72
*General Health Domain*	38.3 ± 23.1	58.7 ± 25.7	0.56	**0.005**	20.3 ± 14.7	**<0.001**	42.7 ± 16.8	34.7 ± 16.0	(−8.0) ± 9.4	**0.005**	50.0 ± 24.2	0.37	13.8 ± 16.7	**0.01**
Physical Function Assessment (FIQ score)	64.2 ± 32.9	34.3 ± 21.0	0.195	**<0.001**	(−29.9) ± 27.3	**0.001**	76.3 ± 10.9	79.5 ± 11.3	3.2 ± 8.4	0.16	38.3 ± 24.2	0.64	(−39.8) ± 21.9	**<0.001**
**BRIEF SYMPTOMS INVENTROY (BSI-18)**														
*Total*	31.4 ± 13.8	14.3 ± 10.6	0.75	**<0.001**	17.1 ± 15.7	**0.001**	33.3 ± 11.1	32.2 ± 11.6	0.53 ± 7.3	0.78	20.5 ± 12.1	0.16	(−12.4) ± 13.0	**0.005**
*Somatization*	9.6 ± 6.3	4.5 ± 4.5	0.35	**<0.001**	5.1 ± 4.9	**0.001**	11.5 ± 4.6	11.0 ± 3.8	(-0.5) ± 3.7	0.59	6.9 ± 4.8	0.18	(−4.1) ± 4.7	**0.009**
*Anxiety*	10.1 ± 4.9	4.3 ± 3.2	0.9	**<0.001**	5.8 ± 5.5	**0.001**	10.3 ± 3.6	11.1 ± 3.8	0.9 ± 3.5	0.36	6.9 ± 3.2	**0.04**	(−4.0) ± 3.7	**0.002**
*Depression*	11.7 ± 5.2	5.6 ± 4.9	0.67	**0.005**	6.1 ± 6.8	**0.004**	11.0 ± 4.0	11.2 ± 5.0	0.2 ± 3.8	0.84	6.6 ± 4.8	0.59	(−4.3) ± 6.2	**0.02**
CTQ	68.9 ± 23.3	66.7 ± 24.3	0.49	0.71	2.3 ± 6.5	0.2	64.2 ± 13.7	63.9 ± 13.7	(−0.1) ± 7.1	0.94	63.4 ± 17.2	0.68	0.15 ± 7.4	0.94
**PSS-I**														
*Total*	28.9 ± 7.4	20.7 ± 9.2	0.73	**0.012**	8.2 ± 9.9	**0.006**	29.9 ± 8.2	28.4 ± 6.4	(−1.4) ± 4.4	0.22	21.8 ± 7.0	0.71	(−5.9) ± 6.2	**0.005**
*Reexperiencing*	7.3 ± 2.8	4.8 ± 3.2	0.6	**0.028**	2.5 ± 3.1	**0.008**	7.9 ± 3.3	7.5 ± 3.1	(−0.4) ± 1.9	0.44	5.8 ± 2.5	0.39	(−1.2) ± 2.1	0.058
*Avoidance*	9.4 ± 2.9	7.5 ± 4.8	0.62	0.14	1.9 ± 4.4	0.11	10.0 ± 3.5	9.6 ± 2.8	(−0.4) ± 2.4	0.53	7.3 ± 2.6	0.92	(−1.8) ± 3.0	**0.05**
*Hyperarousal*	12.2 ± 3.9	8.4 ± 2.8	0.88	**0.008**	3.8 ± 3.8	**0.002**	12.0 ± 3.0	11.3 ± 2.8	(−0.7) ± 2.4	0.31	8.8 ± 2.9	0.74	(−2.9) ± 2.9	**0.003**

*P1 = Baseline scores compared in both groups*.

*P2 = Post scores compared in both groups*.

*P3 = Post score compared to pre score in same group*.

*P4 = Post HBOT scores compared in both groups*.

*P5 = Post HBOT compared to pre-HBOT in control/crossed group*.

*Bold = Significant values, p < 0.05*.

WPI: HBOT effect on participants' pain as reflected by WPI is summarized in Figure S1 and Table 2. After HBOT, the treatment group improved significantly compared to no change in the control group during the control period (*P2* = 0.026). The effect size was large: cohen's D = 1.03. After crossover and HBOT, the control group also improved significantly (*P5* = 0.02, cohen's D = 0.74) with similar mean score as the treatment group.

**Figure 3:**
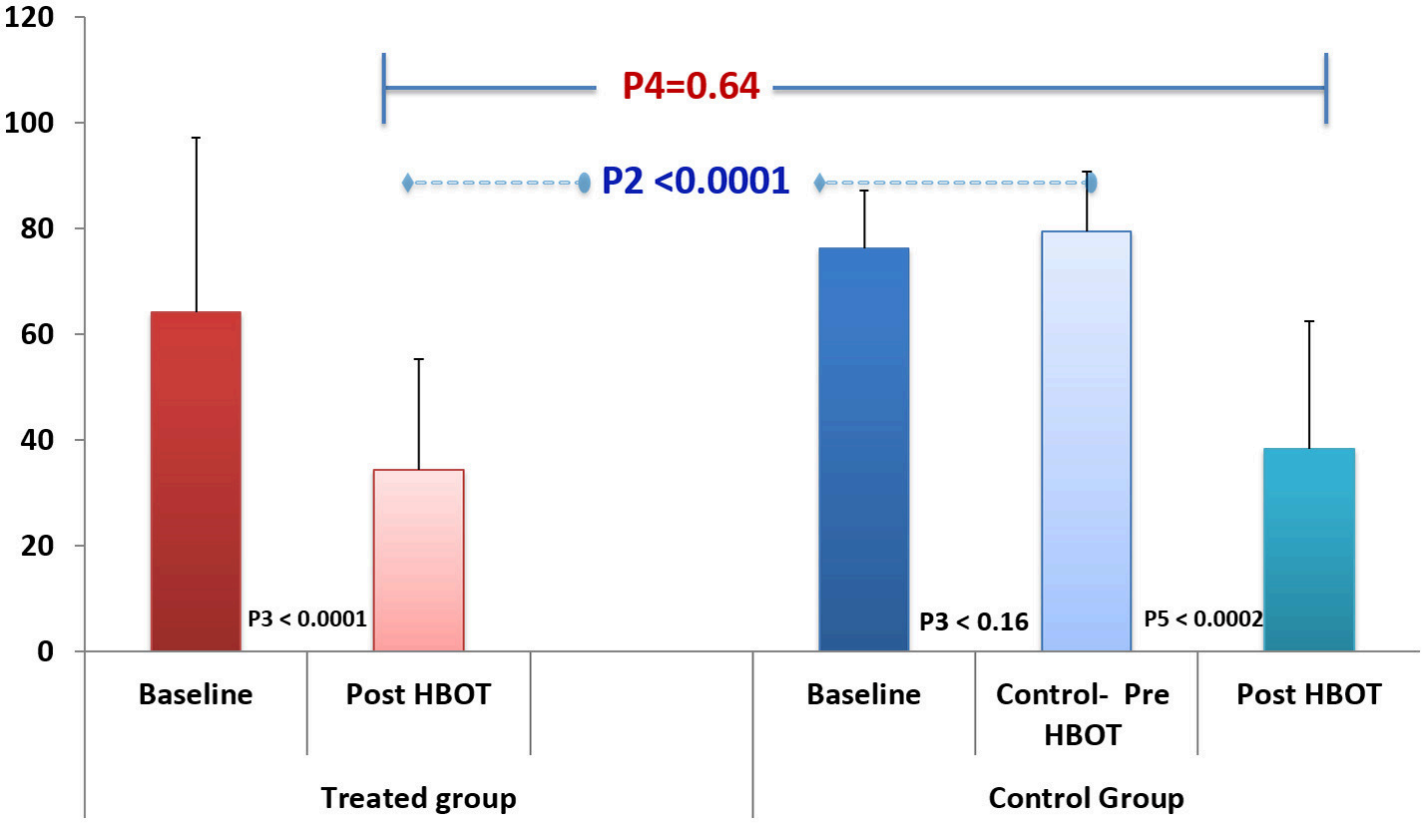
The effect of HBOT on Fibromyalgia impact questionnaire (FIQ).

FIQ: Physical function, measured by FIQ, had similar results as seen in Figure 3 and Table 2; HBOT treatment group had significant improvement from 64.2 ± 32.9 to 34.3 ± 21.0 (*P3* < 0.001, large effect size: cohen's D = 1.32), while the control group had a non-significant negative change of 3.2 ± 8.4 (*P3* = 0.16). Following crossover and HBOT, the control group had a significant improvement of 38.3 ± 24.2 (*P* < 0.001, large effect size, cohen's D = 1.93), similar to the improvement in the treatment group (*P4* = 0.64).

### Effect on Quality of Life

HBOT effect on the SF-36 domains are shown in Table 2 and Figure S2. The treatment group improved in all domains (physical limitations, emotional limitations, energy, emotional well-being, social function, pain, physical function and general) compared to no change or decreased scores following the control period in the control group (*p* < 0.05, large effect size cohen's D > 0.8). After crossover and HBOT, the control group had also significant improvement in most domains of quality of life except pain and emotional limitations.

### Effect on Post Trauma CSA Symptomatology

Somatization, anxiety and depression levels and total score were assessed by the BSI-18 questionnaire are summarized in Table 2 and in Figure S3. HBOT reduced somatization, anxiety and depression levels significantly in more than 50% in the treatment group (*P3* < 0.001) compared to no change in control period following the control period (*P3* > 0.1, between groups *P2* < 0.001). Following crossover and HBOT, the control group improved significantly in all domains (*p* < 0.05).

With regards to PSS-I scores at baseline (*P1* > 0.05), seen in Table 2 and Figure S4, the treatment group improved by 29% after HBOT (*P3* = 0.006) compared to non-significant worsening of 4% in the control group (*P* = 0.22, between groups *P2* = 0.012). Following HBOT, the control group also had a significant improvement of 21% (*P5* = 0.005), comparable to the treatment group.

There were no significant changes in CTQ scores neither in the control period nor post HBOT (Table 2).

### Effect on Brain Activity (SPECT)

**Figure 4:**
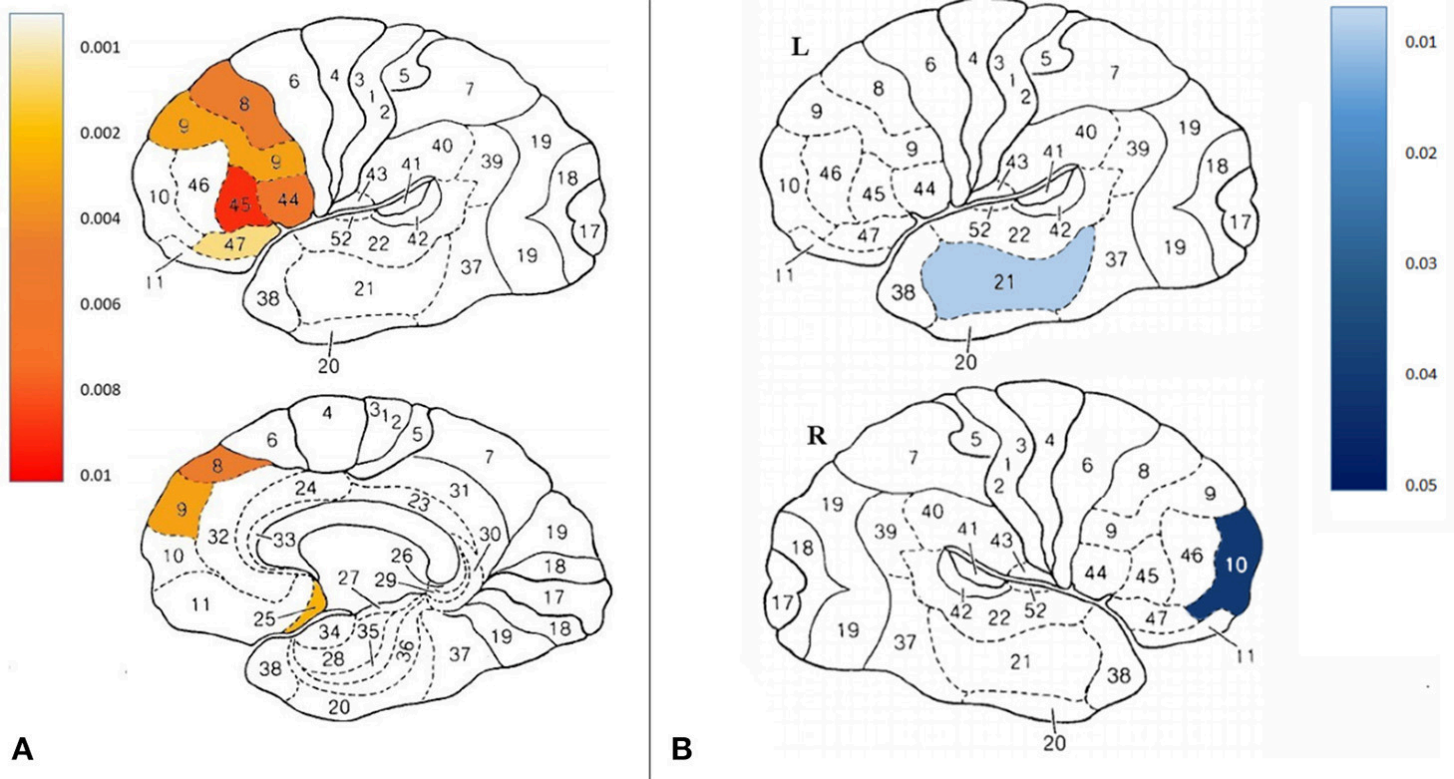
**(A)** Projection of the significant changes between groups on the brain maps **(B)** Projection of the significant changes in the improved compared to non- improved participants on the brain maps.

Fifteen participants in the treatment group and 12 participants in the control group completed all brain SPECT evaluations. Compared to the control group at the control period, there was significant increase in brain activity in the treatment group in the following Broadmann areas (BA): 5R, 8L, 9L, 25L, 44L, 45L, 47L (*p* < 0.05), Figure 4A and Figure S5. Figure 4B demonstrates Broadmann areas with significant decreases in brain activity following HBOT. Following crossover and HBOT, the control group had increase in brain activity in the same areas (*p* < 0.05).

As detailed above, following HBOT, 53% of the participants in the treatment group and 46.7% in the control group recovered and did not fulfill FMS diagnosis criteria. Thus, further investigation was performed to compare brain activity changes between recovered to non-recovered patients. Interestingly, BA10R and BA21L decreased activity significantly in the improved group (*p* < 0.05).

Correlations between perfusion changes and questionnaires scores changes were found significant in the following areas:

- BA5 with physical limitations score (*r* = −0.43, *p* = 0.03)
- BA10 with WPI score (*r* = 0.46, *p* = 0.01) and general health score (*r* = −0.49, *p* = 0.01)
- BA21 with WPI score (*r* = 0.38, *p* = 0.05), physical function score (*r* = −0.41, *p* = 0.04) and general health (*r* = −0.42, *p* = 0.03)
- BA25 with social functioning (*r* = −0.39, *p* = 0.04)
- BA8 with WPI score (*r* = 0.38, *p* = 0.05)
- BA5 with SSS score (*r* = 0.41, *p* = 0.04) and general health score (*r* = −0.43, *p* = 0.02)
- BA44 with FIQ score (*r* = −0.42, *p* = 0.03), BSI total score (*r* = 0.45, *p* = 0.02), physical function score (*r* = −0.4, *p* = 0.04) and general health score (*r* = −0.46, *p* = 0.01)

### Effect on Brain Microstructure

**Figure 5:**
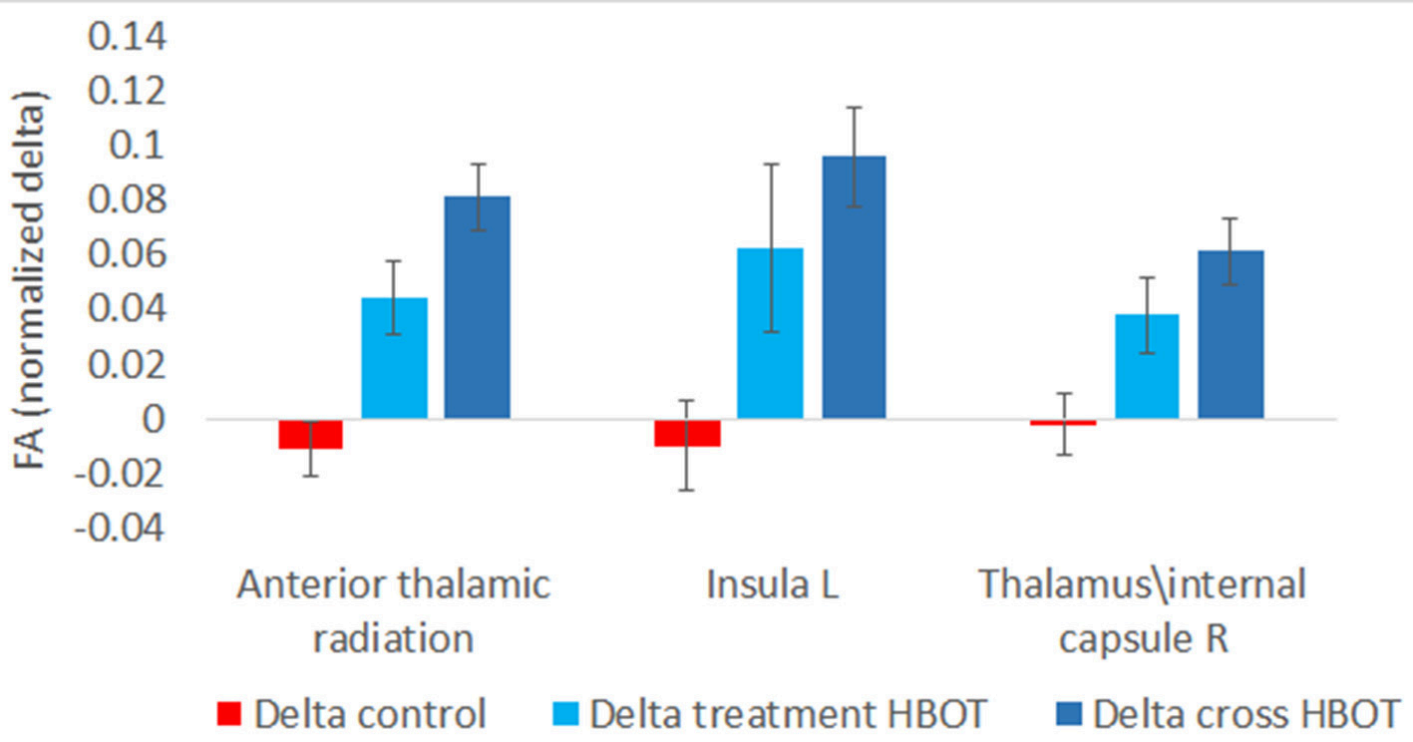
Significant changes in brain microstructure following HBOT.

Fifteen participants in the treatment group and 13 participants in the control group had pre and post MRI evaluations. There was a significant increase in fractional anisotropy (FA) in anterior thalamic radiation, left Insula and right Thalamus and superior thalamic radiation (*p* < 0.001, Figure 5).

Further analysis was performed by grouping the pre-post HBOT changes of all participants (*N* = 27) and compared to the pre-post control period. Post HBOT, there was a significant increase in FA in the same areas: anterior thalamic radiation (*p* < 0.001), left Insula (*p* = 0.001) and the right Thalamus (*p* = 0.001).

### Safety and Other Side Effects

One participant decided to stop HBOT after 20 sessions due to headaches. Twelve participants had mild barotrauma that resolved spontaneously within 2–3 days and did not prevent them from completing the treatment protocol.

Noticeably, 9 participants (32%) reported emotional flooding with re-experiencing childhood events during HBOT. However, all of these participants completed protocol.

## Discussion

The current study presented a prospective active control, clinical trial evaluating the effect of HBOT on women suffering from FMS with a history of CSA at early childhood (mean age 8.4). HBOT induced significant improvement in all FMS measures (WPI, SSS, FMS diagnosis criteria, FIQ) while 53.3% of the treatment group did not fulfill FMS diagnosis criteria after HBTO. In addition, HBOT significantly improved all measures of quality of life (SF-36) and PTSD measures (BSI-18, PSS-I total, somatization and depression but not avoidance). These clinical improvements were accompanied by improvement in brain functionality as evidence by SPECT and brain microstructure as evidence by MRI-DTI. Analysis of the brain functionality by SPECT imaging showed that HBOT induced increased activity in the prefrontal medial cortex (BA8, BA9), pars opercularis and pars triangularis (BA44, BA45), orbital frontal cortex (BA47), and subgenual area (BA25). Brain micostructure analysis, done by MRI-DTI, demonstrated neurogenesis in the anterior thalamic radiation, left Insula, and the right Thalamus.

In the current study, HBOT induced significant changes in the brain metabolism/activity on the following regions:

- **Prefrontal cortex (BA8, BA9)**–The medial prefrontal cortex is one of the brain regions that undergo major developmental changes during childhood and adolescence (Sanchez et al., 2001; Lupien et al., 2009). Animal studies showed prolonged and excessive exposure to stress hormones such as glucocorticoids (as in CSA/PTSD) cause a major change in this area (Sapolsky, 1985). Where CSA survivors show decreased gray matter (GM) volume and medial prefrontal cortex (Andersen et al., 2008; Tomoda et al., 2009; Treadway et al., 2009; Frodl et al., 2010). This prefrontal cortex plays an important role in emotion regulation reduced activity in the left PFC in particular has been associated with negative emotional states (Cardinal et al., 2002; Phillips et al., 2003). Decreased activity/perfusion in the dorsal medial prefrontal cortex has been associated with increased autonomic responsiveness, anxiety, and sad mood (Phillips et al., 2003). HBOT induced significant increase in activity which corresponds with reduced anxiety and depression and regaining emotional control as seen in the BSI-18, PSS-I and SF-36 questionnaires.
- **Orbital frontal cortex (BA47)**–previous study demonstrated reduced activity in this area corresponds with adverse emotional inhibition, social behavioral problems, and norm violations (Berthoz et al., 2002). HBOT increased activity in this area which corresponds to improved SF-36 scores, social function in particular.
- **Subgenual area (BA25)–**This region is a component of the corticolimbic circuits and a component of the inferior portion of anterior cingulate that are disrupted in participants with mood disorders (mainly depression) and has crucial connections to the nucleus accumbens, amygdala, hypothalamus, and orbitofrontal cortex (Yakov Gologorsky and Ron Alterman, 2011). Previous studies showed reduced activity in this area in depression (Niida and Mimura, 2017), FMS patients (Efrati et al., 2015) and PTSD patients (Bremner et al., 1999). Moreover, PTSD patients show significantly decreased blood flow in the subcallosal gyrus (Bremner et al., 1999). HBOT increased activity in this area which corresponds to decreased depression in BSI-18, decreased FMS symptoms in WPI, SSS, FIQ and FMS diagnosis and decreased PTSD symptoms in PSS-I.
- **BA10**–Previous study found increased regional CBF in BA10 in people with major depression (Monkul et al., 2012). In addition, activity in BA10 was increased during performance of a risk-taking task (Rogers et al., 1999). The decrease activity following HBOT may correspond with decreased depression in BSI-18 and increased quality of life (social function) in SF-36.
- **BA21**–The exact function of BA21, known as the middle temporal area, is not fully clear and may be connected with recognition of faces, sounds and other. In a recent study, BA21 activity was markedly increased during recall of a traumatic episode during EMDR sessions (Amano and Toichi, 2016). The reduced activity after HBOT may correspond with reduced re-experiencing in PSS-I.

In addition to normalization of brain activities, HBOT induced significant improvement of brain microstructure in the following areas:

- **Anterior thalamic radiation**–fiber pathways that connect the anterior nuclear group of the thalamus with the frontal lobe, and more specifically to the prefrontal cortex (which was discussed above). Since the anterior thalamic radiation carries fibers from the thalamic nuclei to the prefrontal cortex, it is involved in executive function and planning complex behaviors (Floresco and Grace, 2003). Previous studies have showed reduced FA, a marker of white matter integrity, in the left anterior thalamic radiation (Benedetti et al., 2014) in maltreated children compared to healthy control subjects. HBOT increased FA and improved white matter integrity, which corresponds to prefrontal cortex activation, reduced anxiety and depression and regaining emotional control as seen in the BSI-18, PSS-I, and SF-36 questionnaires.
- **Thalamus**–The thalamus is a key relay station for the transmission of nociceptive information to the cerebral cortex. Nociceptive inputs from the skin, deep structures, and visceral organs converge in the thalamus a route to the cerebral cortex. Due to its reciprocal connection with the cerebral cortex, the thalamus is a key player of chronic pain conditions (Yen and Lu, 2013). Patients suffering from chronic spontaneous pain show altered regional CBF in the thalamus (Linnman et al., 2009). fMRI studies demonstrated FMS patients had a reduced pain related activation of thalamus compared with healthy controls (Jensen et al., 2009). MRI-DTI studies have showed significant decreased FA in both thalami, the thalamocortical tracts, and both insular regions (Lutz et al., 2008). HBOT induced increased FA in the thalamus, which corresponds to reduced pain and FMS symptoms in WPI, SSS, FIQ, and FMS diagnosis criteria.
- Apart from the thalamus relation to pain, previous studies showed decreased thalamic perfusion in PTSD (Kim et al., 2007), decreased thalamic activation during integral generation of memories of the traumatic event in adults with PTSD in fMRI (Lanius et al., 2003). In addition, decreased mean diffusivity in MRI-DTI was found in children with PTSD (Lei et al., 2015). HBOT increased FA in the thalamus, which may correspond with decreased PSS-I scores and anxiety scores in BSI-18.
- **Insula**–The insula is a cortical structure buried within the Sylvian fissure behind the superior temporal lobe which is crucial for the representation and processing of internal bodily signals thought to form the basis for subjective emotional states (Craig, 2009). PTSD patients have reduced functional connectivity compared with healthy controls between the left ventral anterior insula and the anterior cingulate cortex. PTSD patients also exhibit decreased functional connectivity between the right posterior insula and left inferior parietal lobe, and the postcentral gyrus (Zhang et al., 2016). In addition, CSA correlated with connectivity in and among the insula, anterior cingulate cortex, and prefrontal cortices—which are brain structures that are important for processing, expression, and regulation of emotional states (Craig, 2009). HBOT increases FA in the insula and improves its connectivity, and may respond to reduced PSS-I, BSI-18 and SF-36 scores.

FMS symptoms improvements, evident at this study, stand in line with previous studies by Yildiz and Efrati who demonstrated the effect of HBOT on FMS pain and quality of life in FMS from different etiologies (Yildiz et al., 2004; Efrati et al., 2015). However, the unique population of CSA women in this study who failed all other treatments, was treated for the first time with biological intervention that induces neuroplasticity–HBOT. Although there is a substantial amount of literature evaluating efficacy of different medications and psychotherapy approaches in FMS and CSA, most of them are of low level of evidence, symptoms-targeted and did not use any neuroimaging (Seligman, 1995; Callahan et al., 2004; Martsolf and Draucker, 2005; Bisson et al., 2007; Ehring et al., 2014). In this study, for the first time both symptoms and quality of life improvements were correlated with the improvement in brain performance parameters as seen in brain function (SPECT) and structure (MRI-DTI) imaging. The current study results stand in line with the new understanding by which FMS pathophysiology is an abnormal pain transmission and processing within the central nervous system (Ablin et al., 2016).

Unlike psychotherapy or pharmacotherapy approaches, HBOT is a physiological/biological therapeutic intervention with direct effects on brain functionality (blood flow and repair processes). HBOT may enable the metabolic change by supplying the energy needed for these repair processes (Efrati et al., 2013; Efrati and Ben-Jacob, 2014). Recent studies provided convincing evidence that HBOT could induce neuroplasticity in humans leading to repair of chronically impaired brain functions and improved quality of life in post stroke patients, post TBI patients and recently, FMS patients (Boussi-Gross et al., 2013, 2015; Efrati et al., 2015). As demonstrated above, these mechanisms enable HBOT to induce the neuroplasticity needed to overcome both abnormal function and microstructural organization which translate to significant clinical improvement in all FMS and PTSD symptoms, as well as significant improvement of quality of life.

A supportive clinical observation for the notion that HBOT is indeed inducing neuroplasticity can be drawn from the qualitative findings, where most of the participants reported worsening of their symptoms during first 20 sessions (stage 1). Same worsening was also reported in the previous HBOT study done on FMS (Efrati et al., 2015). The symptoms worsening during the first sessions may be explained due to HBOT-induced metabolic and circuitry changes in brain areas associated with emotional and pain processing.

### Study Limitations

First, the sample size of *N* = 30 is rather small. Obviously, larger scale clinical trials are required to reconfirm the findings presented.

Second, even with randomization and blinding in analysis, there was no blinding performed to the participants due to the inherent difficulty conducting sham control in HBOT trials (Efrati and Ben-Jacob, 2014) (For further discussion on the control/placebo dilemma in this study see Appendix-III). This could be a possible effector on the questionnaires. However, the correspondence between the clinical improvement and brain functional and structural improvements as evident by the brain imaging, substantiates the clinical findings. Moreover, the association between the anatomical locations of the changes in the brain function, as demonstrated by the SPECT, and brain structure as demonstrated by MRI-DTI, and the clinical findings provide important validation of the evaluation. In addition, the symptoms worsening during the first 20 sessions, unexpected by the patients, warrant that there is indeed direct biological effect of HBOT on this cohort of FMS patients.

### Strengths

1. The study was conducted as a randomized prospective crossover trial. Strict inclusion was performed using the new 2010 FMS criteria (Wolfe et al., 2011).
2. The questionnaires were performed in an individual computerized blinded method.
3. For the first time in clinical studies on FMS who are CSA survivors, both functional and anatomical (microstructure) brain imaging were performed to validate the clinical effects.
4. Strict statistical analysis was performed including false discovery rate correction for multiple comparisons on brain imaging in order to avoid misleading results “by-chance.”

## Conclusion

This study provides evidence that HBOT can induce a significant improvement in patients suffering from FMS with a history of CSA. HBOT induced neuroplasticity and significantly improve both brain function and brain microstructure in CSA related areas. Further studies are required to find the optimal dose-response curve and optimal time of treatment.

## Author Contributions

SE, YB, and RL-W Conceived and designed the experiments. AH, YB, SD-T, TS, MCa, MCo, and SE Performed the experiments. AH, YB, SE, MCa, and RL-W Analyzed the data. AH, YB, SE, SD-T, MCa, and RL-W Wrote the paper.

### Conflict of Interest Statement

The authors declare that the research was conducted in the absence of any commercial or financial relationships that could be construed as a potential conflict of interest.
