# Supplementary material for: Hyperbaric Oxygen Therapy Can Induce Neuroplasticity and Significant Clinical Improvement in Patients Suffering From Fibromyalgia With a History of Childhood Sexual Abuse—Randomized Controlled Trial
Source: Front Psychol. 2018 Dec 17;9:2495. doi: 10.3389/fpsyg.2018.02495 (PMC6304433; doi:10.3389/fpsyg.2018.02495)
Supplement: Supplementary file 1 [file Data_Sheet_1.docx]

Supplementary materials

Sham/Placebo Dilemma

The researchers did not utilize a sham/placebo in the control group for several reasons. First, there are inherent ethical and logistic difficulties in handling the sham-control in HBOT trials ^1-4^. The standard requirement from proper sham-control is: “Medically ineffectual treatment for medical conditions intended to deceive the recipient from knowing which treatment is given”. Hyperbaric oxygen therapy includes two active ingredients: pressure and oxygen. The pressure is being utilized for increasing plasma oxygen, but the pressure change by itself may have significant effects on the cellular level. The pressure effect may be of greater significance in human tissues that are under tight autoregulation pressure control, such as the brain and kidneys^5-9^. To generate the pressure sensation, the chamber pressure must be 1.2ATA or higher. However, that significant change in the environmental pressure cannot be referred as sham but rather relative low dose of the active ingredient^2,4^.

Second, this study's population of patients with fibromyalgia induced by childhood sexual abuse, added further complexity to this decision. During HBOT sessions, these patients suffer from both physical difficulties (such as pain or considerate discomfort) and emotional overload (such as outbursts, crying, re-experiencing repressed conscious or unconscious memories).

Third, prior to their inclusion, all patients had failed different psychological and pharmacological interventions (the mean age at inclusion was 45.9±10.8 and the sexual assault was at the of 8.4±4.5 years. Since these unfortunate patients exhausted all available therapeutic interventions, the hyperbaric oxygen therapy can be considered as a salvage therapy for unremitting long standing debilitating disease.

Taking it all together, the researchers came to a conclusion that 60 sessions of sham treatment with the considerable constrains related to this study population would be difficult as well as unethical.Thus. the cross-over designed was chosen. These considerations and the cross over design were approved by the institutional Helsinki ethic committee.

Appendix-III E-Figures

Figure-S1: The effect of HBOT on Widespread pain index (WPI)

| 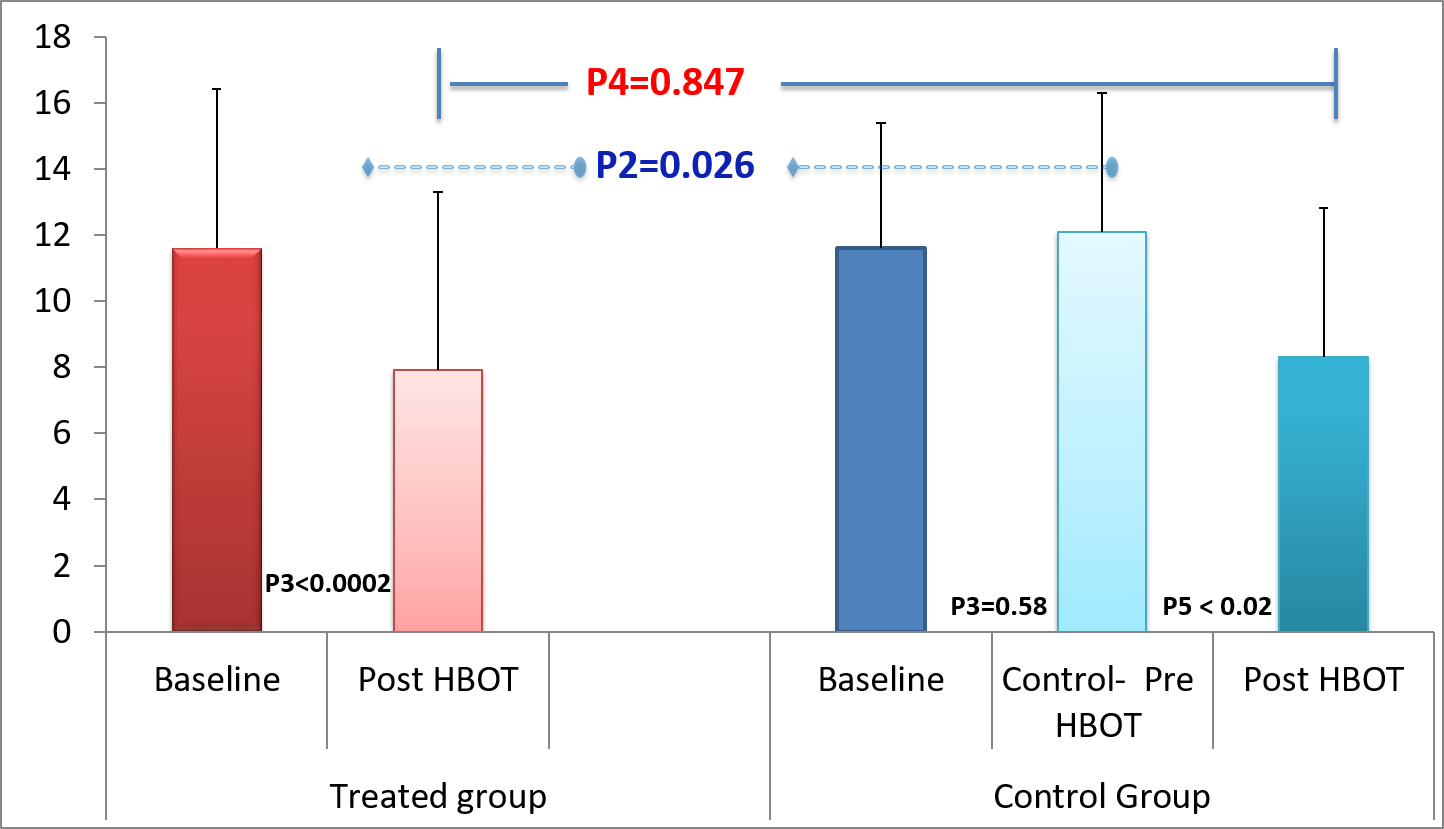  P1=Baseline scores compared in both groups. |
| --- |
| P2=Post scores compared in both groups. |
| P3=Post score compared to pre score in same group. |
| P4=Post HBOT scores compared in both groups. |
| P5=Post HBOT compared to pre-HBOT in control/crossed group |

Figure-S2: The effect of HBOT on SF-36 general domain


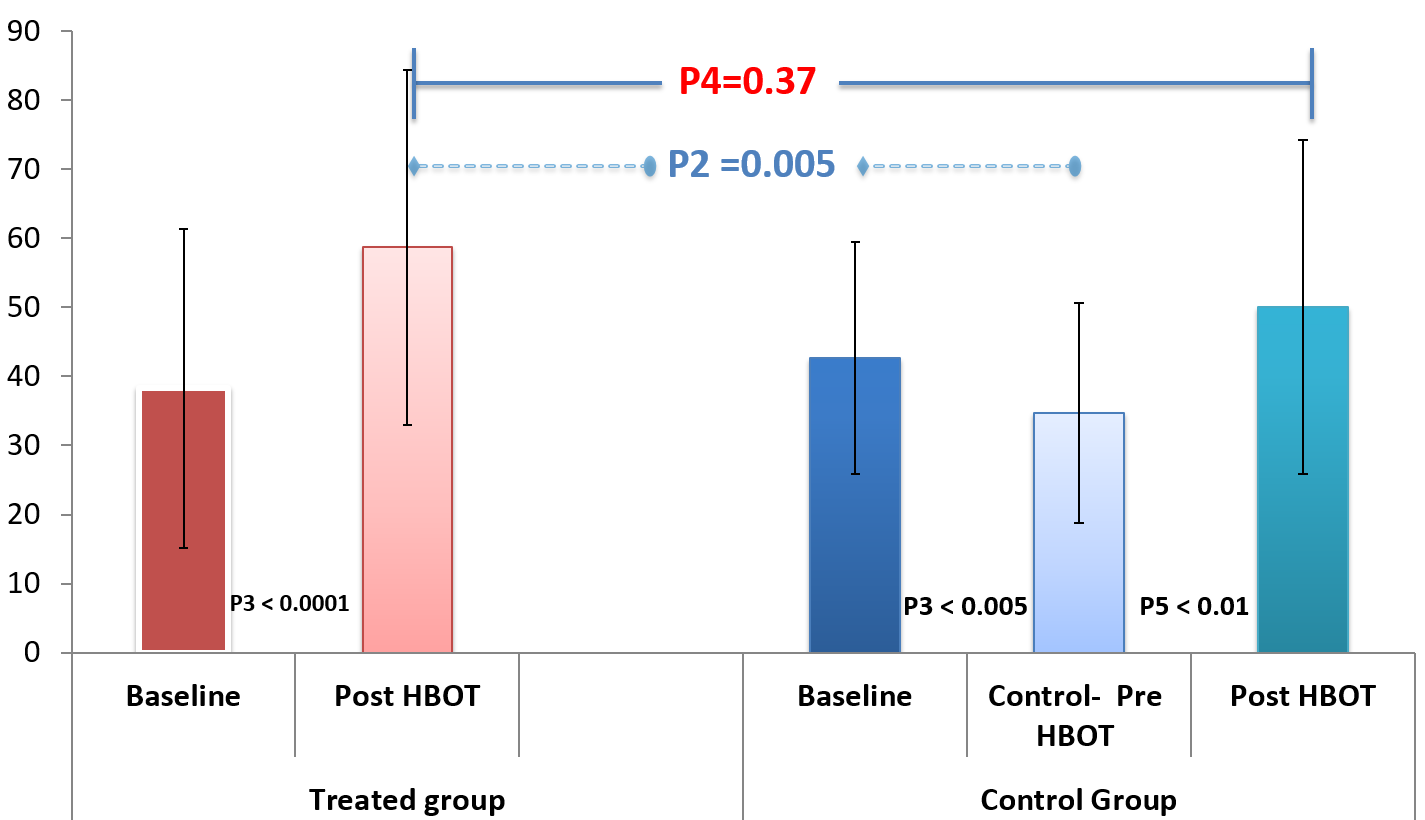


Figure-S3: The effect of HBOT on BSI-18 domains
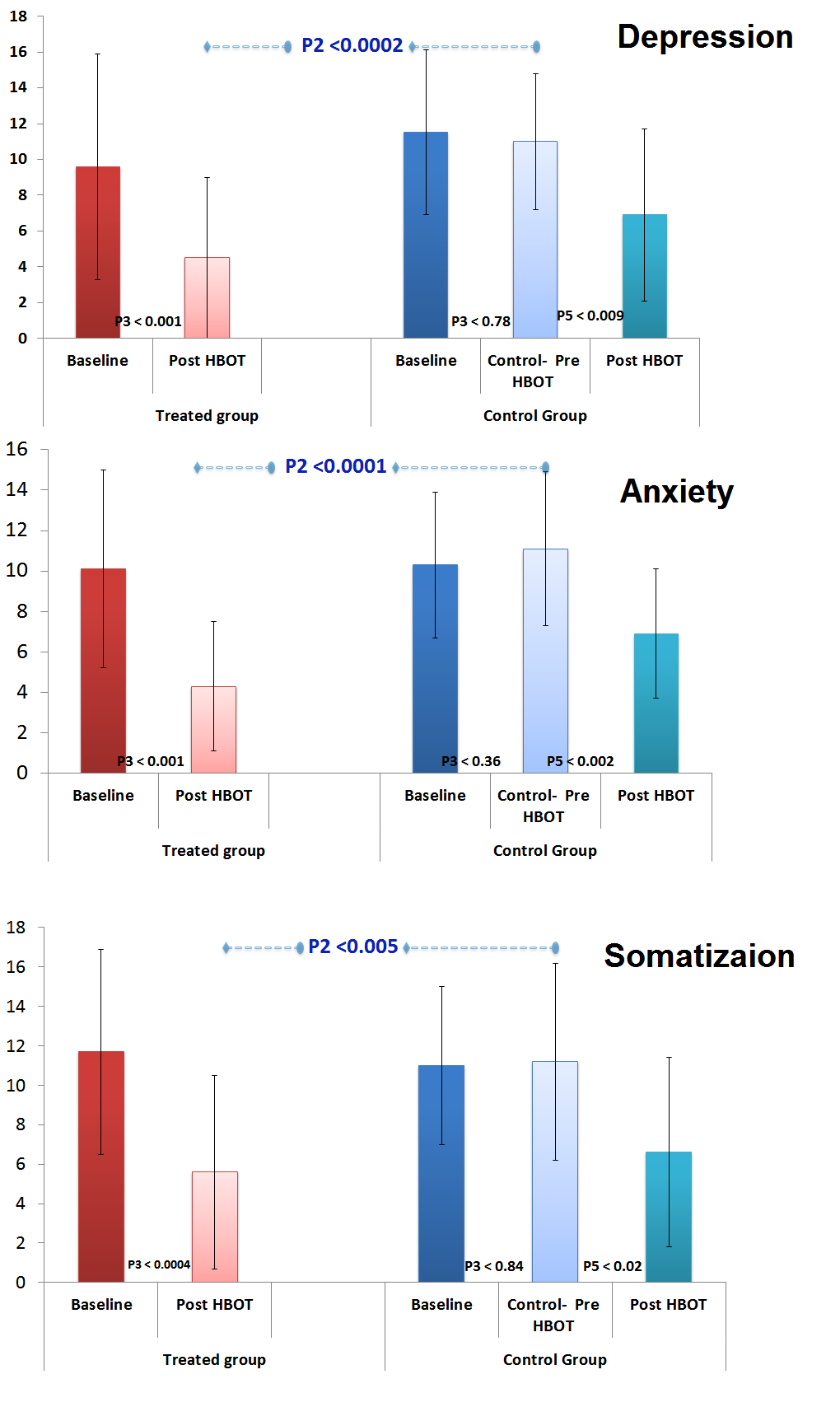


Figure-S4: The effect of HBOT on PSS-I total score


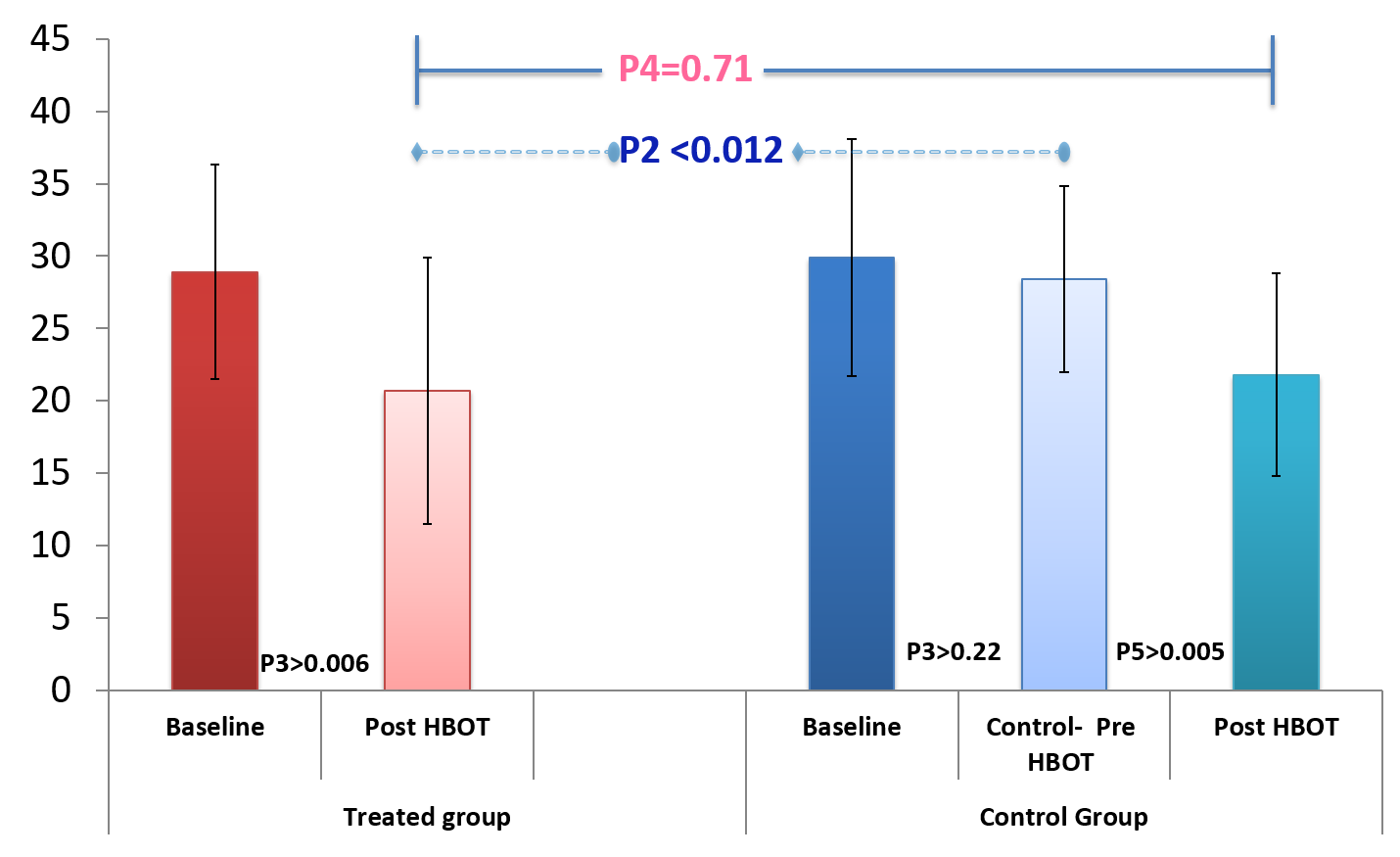


Figure-S5: Box plot analysis of the mean percent of relative change in SPECT activation during the three experimental phases.


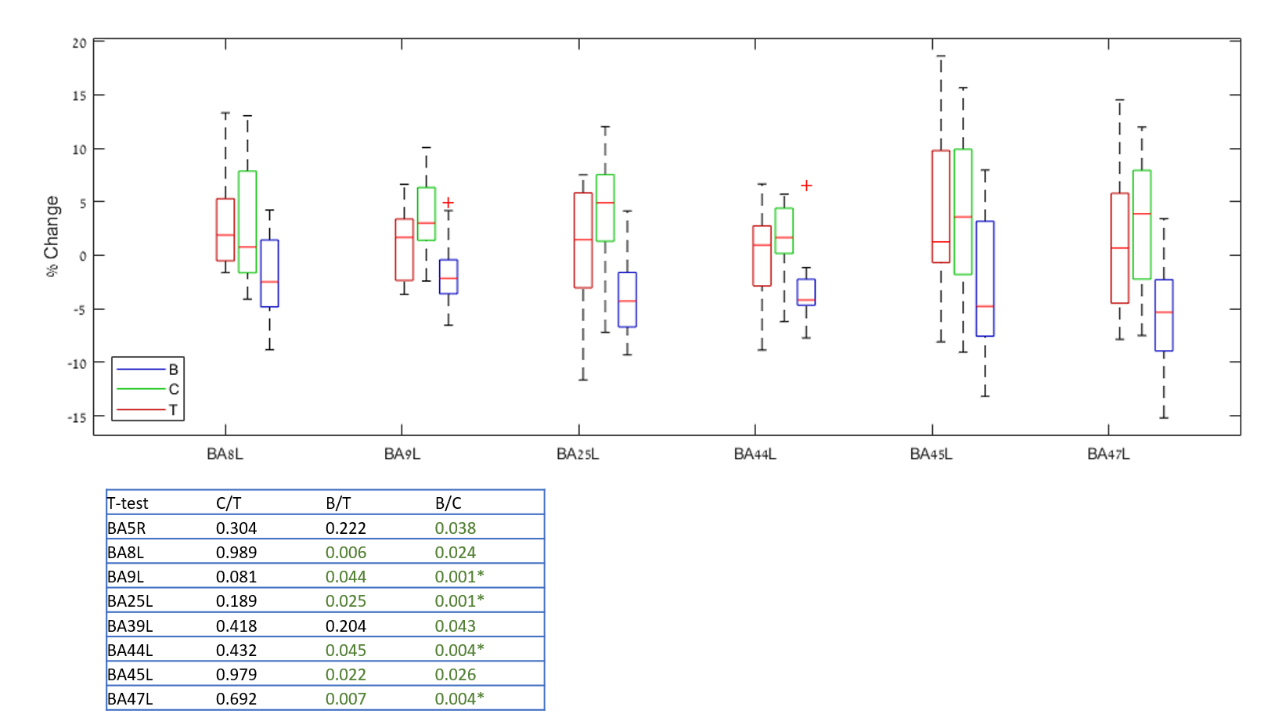


Significant changes in brain activity in SPECT analysis of the 2 groups

* FDR (P < 0.05 corrected)

Table S-6: Significant correlations between Brodmann areas perfusion and questionnaires scores

| **BA** | **Questionnaire** | **After HBOT** | | **After Control period** | |
| --- | --- | --- | --- | --- | --- |
|  |  | **r** | **p** | **r** | **p** |
| **BA5** | Physical limitations | -0.43 | 0.03 | 0.41 | 0.19 |
|  | SSS | 0.41 | 0.04 | -0.17 | 0.59 |
|  | General health | -0.43 | 0.02 | 0.43 | 0.16 |
| **BA8** | WPI | 0.38 | 0.05 | -0.34 | 0.28 |
| **BA10** | WPI | 0.46 | 0.01 | 0.31 | 0.33 |
|  | General health | -0.49 | 0.01 | 0.25 | 0.42 |
| **BA21** | WPI | 0.38 | 0.05 | 0.19 | 0.55 |
|  | Physical function | -0.41 | 0.04 | -0.35 | 027 |
|  | General health | -0.42 | 0.03 | 0.21 | 0.5 |
| **BA25** | Social functioning | -0.39 | 0.04 | -0.16 | 0.62 |
| **BA44** | FIQ | -0.42 | 0.03 | 0.49 | 0.11 |
|  | BSI total | 0.45 | 0.02 | -0.30 | 0.34 |
|  | Physical function | -0.4 | 0.04 | -0.43 | 0.15 |
|  | General health | -0.46 | 0.01 | -0.48 | 0.11 |

BA=Brodmann area

r= correlation coefficient

p=pvalue

On the left, significant correlations between change in BA perfusion and questionnaire scores. Whereas on the right, no significant correlations in changes after the control period (without HBOT).

References

1. Efrati S, Ben-Jacob E. How and why hyperbaric oxygen therapy can bring new hope for children suffering from cerebral palsy--an editorial perspective. *Undersea & hyperbaric medicine : journal of the Undersea and Hyperbaric Medical Society, Inc.* Mar-Apr 2014;41(2):71-76.

2. Figueroa XA, Wright JK. Hyperbaric oxygen: B-level evidence in mild traumatic brain injury clinical trials. *Neurology.* Sep 27 2016;87(13):1400-1406.

3. Efrati S, Ben-Jacob E. Reflections on the neurotherapeutic effects of hyperbaric oxygen. *Expert Rev Neurother.* Mar 2014;14(3):233-236.

4. Hadanny A, Efrati S. Treatment of persistent post-concussion syndrome due to mild traumatic brain injury: current status and future directions. *Expert Rev Neurother.* Aug 2016;16(8):875-887.

5. Etzion Y, Grossman Y. Pressure-induced depression of synaptic transmission in the cerebellar parallel fibre synapse involves suppression of presynaptic N-type Ca2+ channels. *Eur J Neurosci.* Nov 2000;12(11):4007-4016.

6. Hanlo PW, Gooskens RJ, van Schooneveld M, et al. The effect of intracranial pressure on myelination and the relationship with neurodevelopment in infantile hydrocephalus. *Dev Med Child Neurol.* May 1997;39(5):286-291.

7. Berman S, Abu Hamad R, Efrati S. Mesangial cells are responsible for orchestrating the renal podocytes injury in the context of malignant hypertension. *Nephrology (Carlton).* Apr 2013;18(4):292-298.

8. Efrati S, Berman S, Goldfinger N, et al. Enhanced angiotensin II production by renal mesangium is responsible for apoptosis/proliferation of endothelial and epithelial cells in a model of malignant hypertension. *J Hypertens.* May 2007;25(5):1041-1052.

9. Johnson W, Nguyen ML, Patel R. Hypertension crisis in the emergency department. *Cardiol Clin.* Nov 2012;30(4):533-543.
